# Widely Targeted Metabolomic Analysis of Metabolic Differences Among Various Organs of *Clematis huchouensis* from the Huzhou Production Region

**DOI:** 10.3390/metabo16080561

**Published:** 2026-08-09

**Authors:** Minyan Song, Yan Yang, Guoping Ni, Yan Zhang, Yiming Zhang, Lixia Zhou, Junhong Zhang

**Affiliations:** 1 Huzhou Vocational & Technical College, Huzhou 313000, China; 2023065@huvtc.edu.cn (M.S.); 2014015@huvtc.edu.cn (Y.Z.);; 2 Zhejiang Key Laboratory of Forest Genetics and Breeding, College of Forestry and Biotechnology, Zhejiang A&F University, 666 Wusu Street, Lin’an, Hangzhou 311300, China

**Keywords:** *Clematis huchouensis*, leave, stem, root, widely targeted metabolomic

## Abstract

Background/Objectives: *Clematis huchouensis* Tamura, a genuine medicinal herb endemic to Huzhou, Zhejiang Province. However, its secondary metabolic profile and organ-specific distribution of bioactive constituents remain largely uncharacterized. This study aims to systematically characterize the metabolic profile of its roots, stems, and leaves, and to elucidate organ-specific accumulation patterns of pharmacologically relevant constituents, thereby providing a scientific basis for resource evaluation and quality control of this regional germplasm. Methods: A widely targeted metabolomics approach was employed to profile metabolites in the roots, stems, and leaves of *C. huchouensis*. Comprehensive annotation and relative quantification were performed using ultra-performance liquid chromatography–tandem mass spectrometry (UPLC-MS/MS) combined with database matching. Cluster analysis and pathway enrichment were conducted to compare metabolic profiles across organs. Results: A total of 1561 metabolites were identified, exhibiting distinct organ-specific accumulation patterns. Flavonoids, alkaloids, and most phenolic acids were predominantly enriched in the aerial parts, whereas the roots accumulated high levels of glutathione and its related peptides, reflecting their significant antioxidant activity. Amino acids displayed complementary tissue-specific distribution, with peptides enriched in leaves and sulfur-containing amino acids such as L-methionine in stems. Organ-specific metabolic differences were significantly associated with pathways including flavonoid biosynthesis and linoleic acid metabolism. Notably, numerous pharmacologically active compounds, such as hispidulin, diosmetin, trigonelline, colchicoside, and oleanolic acid-3-*O*-xylosyl(1→3)glucuronide—exhibited marked tissue-selective accumulation. Conclusions: This first metabolomic study of *C. huchouensis* reveals organ-specific accumulation of bioactive compounds, providing a metabolic foundation for its quality control and rational utilization.

## 1. Introduction

*Clematis* belongs to the Ranunculaceae family and is a perennial liana genus with more than 350 species [1,2]. The genus *Clematis* is primarily distributed in temperate and subtropical regions, with particularly abundant germplasm resources in East Asia [1]. China represents a major distribution center for the genus *Clematis*, with over 150 species having been documented within its territory [3,4]. Many species within the genus *Clematis* are valued as ornamental plants in horticulture due to their large and showy flowers. Notable examples include *C. vitalba*, *C. lanuginosa*, and *C. hancockiana* [5,6]. Furthermore, as an important group of medicinal resources, plants of the genus *Clematis* have been studied and utilized worldwide due to their diverse pharmacological activities [7]. A prominent example is the traditional Chinese medicine “Wei Ling Xian,” prepared from the dried roots and rhizomes of *C. chinensis* Osbeck, *C. hexapetala* Pall., or *C. manshurica* Rupr. This herbal remedy has been traditionally employed as an analgesic, diuretic, and antitumor agent, and is particularly noted for its efficacy in treating rheumatoid arthritis and various inflammatory diseases [6]. With the in-depth advancement of phytochemical and pharmacological research in recent years, researchers have gradually clarified a variety of bioactive constituents contained in *Clematis* species and revealed their corresponding action mechanisms. For instance, the roots of *C. chinensis* are rich in saponins and pectin-like polysaccharides. Mechanistic studies have demonstrated that its saponins alleviate osteoarthritis by inhibiting mitochondrial damage and caspase-3 activation [8,9], and the pectin-like polysaccharides alleviate rheumatoid arthritis by suppressing the expression of pro-inflammatory cytokines and matrix metalloproteinase genes [10]. Similarly, clematichinenoside, a compound isolated from the roots of *C. hexapetala*, exhibits potent immunomodulatory activity. This effect is mediated by the downregulation of TNF-α levels, highlighting its therapeutic potential in chronic inflammation [11]. Further studies have revealed anti-neuroinflammatory properties associated with phenolic compounds and lignans derived from the rhizomes of *C. manshurica* [12,13]. Triterpenoid saponins from the roots of *C. uncinata* have demonstrated significant antitumor activity [14], while a novel cyclopeptide alkaloid and coumarins isolated from the roots of *C. florida* possess anti-inflammatory properties [15]. Beyond applications for internal disorders, the leaves of *C. flammula* have been traditionally used for the topical treatment of superficial skin burns [16].

**Figure 1:**
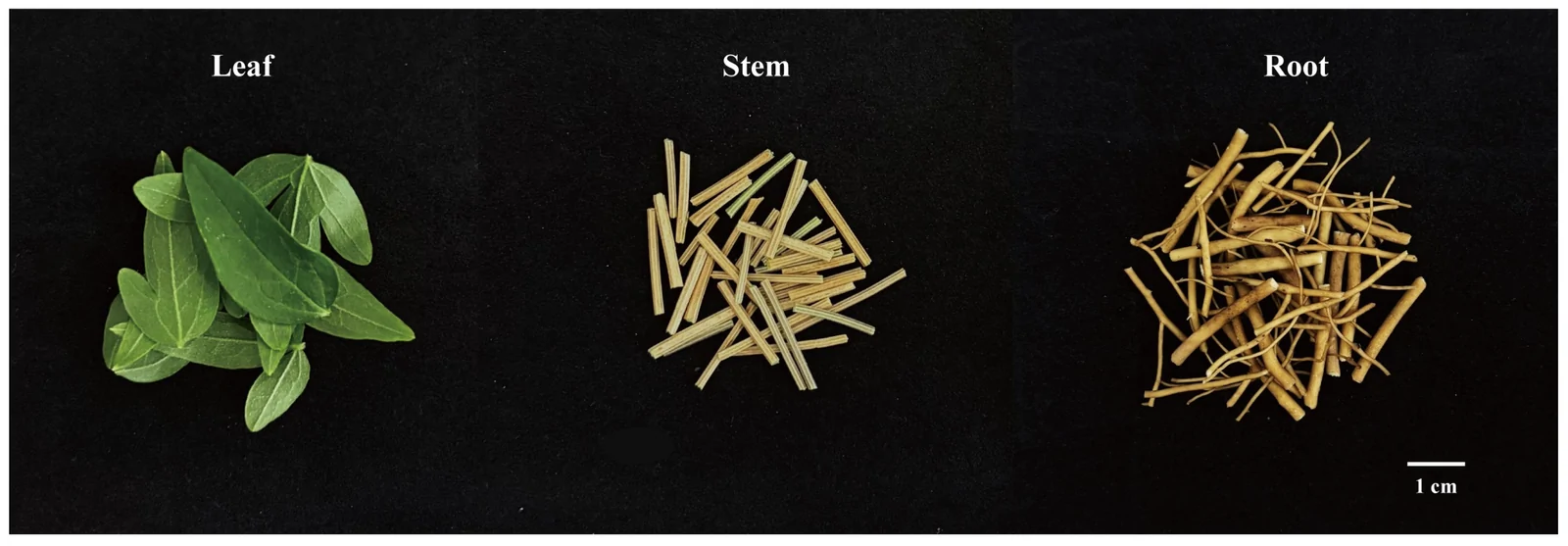
Morphological illustration of the root, stem, and leaf of *C. huchouensis*.

*C. huchouensis*, also known as “Riverside Wei Ling Xian”, is a genuine medicinal herb native to Huzhou, Zhejiang Province, and its whole plant is used for medicinal purposes (Figure 1). *C. huchouensis*-based traditional Chinese medicine preparations are widely used clinically for treating rheumatoid arthritis [2]. Moreover, in traditional Chinese medicine practice, its combination with other herbs yields multiple bioactivities, including anti-tumor, anti-inflammatory, and anti-allergic properties in traditional Chinese medicine practice. In traditional folk practice, the herb is commonly pounded and applied topically or boiled into a decoction for oral intake to alleviate pain and address deep-seated abscesses as well as brain tumor-associated manifestations. Meanwhile, it has also found clinical application in the management of toothache, gout, hepatic carcinoma, and lymphadenitis [1,3]. Studies have shown that the polysaccharide extracted from *C. huchouensis* (CP), as an acidic proteoglycan, exhibits significant scavenging activity against superoxide anions and hydroxyl radicals in vitro, along with strong reducing power, suggesting its promising potential for antioxidant applications.

As a regionally distinctive germplasm, *C. huchouensis* holds a unique position in local ecosystems and medicinal applications. However, its secondary metabolic characteristics, material basis, and patterns of organ-specific accumulation remain poorly understood. Here, we performed the first systematic analysis using widely targeted metabolomics technology to investigate the metabolite composition and differences across various organs of *C. huchouensis*, thereby elucidating its metabolomic profile. This discovery fills a gap in metabolomic research on this species, enriches the database for the genus *Clematis*, and provides a novel metabolic-level basis for studies on phylogenetic evolution, species identification, and resource evaluation within the genus.

## 2. Materials and Methods

### 2.1. Plant Material

The *C. huchouensis* specimen was collected in June 2025 along a riverside in Huzhou City, Zhejiang Province, China. The *C. huchouensis* growth conditions are consistent with the external environment at the place of origin. The *C. huchouensis* specimen was collected in June 2025 from Longxi River (Longxi Port, West Tiaoxi) in Huzhou City, Zhejiang Province, China. The geographic coordinates of the sampling location are 120.0252° E, 30.8855° N, and the habitat is riparian wetland along the riverbank. The growth conditions of *C. huchouensis* match the ambient environment of its native habitat. Three *C. huchouensis* plants with good growth conditions were randomly selected to obtain three organs: roots, stems, and leaves. They were immediately frozen in liquid nitrogen and placed overnight at −80 °C before being transferred to a pre-cooled freeze dryer (FD-250201GX, FTFDS, Shanghai, China) for freeze-drying.

### 2.2. Sample Extraction

After freeze-drying, the sample was ground at 50 Hz for 40 s using a grinder (MM 400, Retsch GmbH, Haan, Germany). A 50 mg aliquot of the powder was mixed with 1.2 mL of methanol/water (4:1, *v*/*v*) containing an internal standard. The mixture was vortexed and shaken for 30 s, homogenized at 35 Hz for 5 min, and then sonicated in an ice-water bath for 4 min. This homogenization-sonication cycle was repeated three times. The mixture was subsequently kept at 4 °C for 1 h. After centrifugation at 12,000 r/min for 5 min, the supernatant was filtered through a 0.22 μm membrane and transferred into an autosampler vial. Quality control (QC) samples were prepared by pooling equal volumes of each sample for subsequent analysis.

### 2.3. Ultra-Performance Liquid Chromatography Conditions

The sample extracts were analyzed using a UPLC-ESI-MS/MS system, comprising an UPLC system (ExionLC^TM^ AD, SCIEX, Singapore) and a triple quadrupole-linear ion trap (QTRAP 6500, SCIEX, Singapore) mass spectrometer (both from Sciex). Chromatographic separation was performed on an Agilent SB-C18 column (1.8 µm, 2.1 mm × 100 mm, Agilent Technologies, Santa Clara, CA, USA) at 40 °C, with a flow rate of 0.35 mL/min and an injection volume of 2 µL. The mobile phase consisted of 0.1% formic acid in water (A) and 0.1% formic acid in acetonitrile (B). Separation was achieved using a gradient program: initial conditions of 95% A and 5% B were maintained, followed by a linear gradient to 5% A and 95% B over 9 min, held for 1 min, then returned to 95% A and 5% B within 1.1 min and equilibrated for 2.9 min. The column effluent was introduced into the ESI-QTRAP-MS for detection.

### 2.4. ESI-Q TRAP-MS/MS

The operating parameters for the ESI source were: temperature, 500 °C; ion spray voltage, ±5500 V (positive)/−4500 V (negative); GS1, GS2, and curtain gas pressures at 50, 60, and 25 psi, respectively; and CAD gas set to high. All QQQ scans were acquired using MRM mode, with the collision gas (nitrogen) pressure set to medium. The declustering potential and collision energy for each individual MRM transition were determined through separate optimization. For each acquisition period, a specific set of MRM transitions was monitored, based on the elution profiles of the target metabolites within that interval.

### 2.5. Metabolite Identification and Quantification

Metabolites were identified by matching secondary mass spectrometry data against an in-house metabolite database. Prior to analysis, the data were preprocessed to remove isotopic signals, redundant signals caused by K^+^, Na^+^, and NH_4_^+^ adducts, as well as fragment ions derived from higher molecular weight metabolites. For quantification on a triple quadrupole mass spectrometer, multiple reaction monitoring mode was employed. This mode operates by selectively filtering the precursor ion of interest with the first quadrupole, fragmenting it in the collision cell, and detecting a specific product ion with the third quadrupole. After obtaining mass spectrometry data from all samples, chromatographic peaks of each metabolite were integrated. Peak alignment and integration correction were applied to the same metabolite across different samples based on retention time to ensure data consistency.

### 2.6. Data Analysis

Unsupervised principal component analysis (PCA) was conducted using the prcomp function in R (www.r-project.org), with unit variance scaling applied to the data prior to analysis. Pearson correlation coefficients (PCC) between samples were calculated by the cor function in R and presented as only heatmaps. The identification of differential metabolites was based on different thresholds depending on the group design. For two-group analyses, metabolites with VIP > 1 and |Log2FC| ≥ 1.0 were considered differential. For multi-group analyses, the criteria were VIP > 1 and ANOVA *p*-value < 0.05. The OPLS-DA model, from which VIP values and the score/permutation plots were generated, was constructed using the MetaboAnalystR package in R. The input data were log2-transformed and mean-centered before modeling. A permutation test (*n* = 200) was additionally performed to assess model validity and prevent overfitting. Identified metabolites were annotated using the KEGG Compound database (http://www.kegg.jp/kegg/compound/ accessed on 27 August 2025), and then mapped to the KEGG Pathway database (http://www.kegg.jp/kegg/pathway.html accessed on 10 September 2025).

## 3. Results

### 3.1. Total Ion Current Diagram Analysis

**Figure 2:**
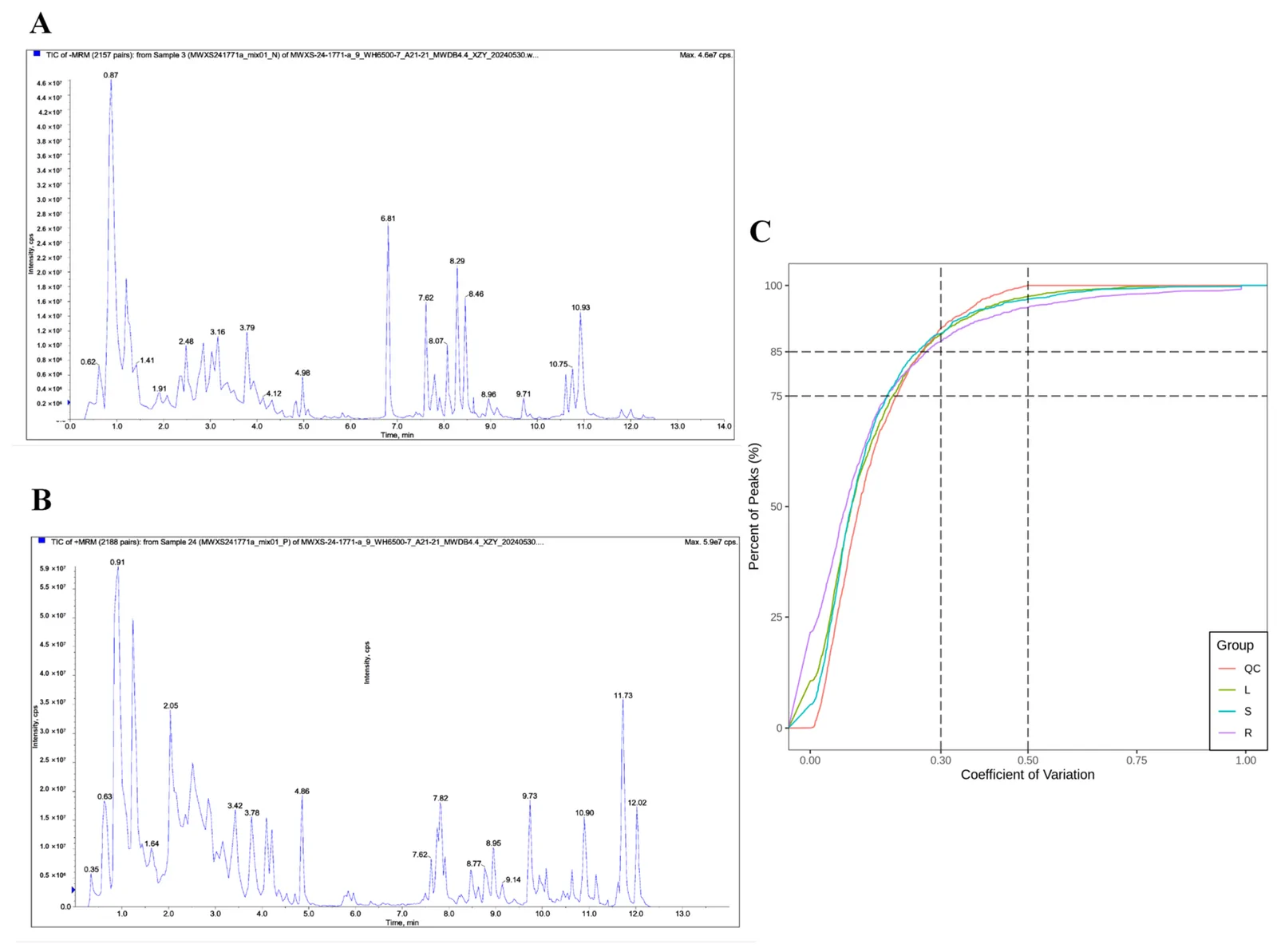
Overlay of total ion chromatograms from QC samples. (**A**): Negative ion mode; (**B**): Positive ion mode; (**C**): Distribution of CV values in QC samples.

Nearly perfect overlap was observed in the total ion chromatograms (TIC) of QC samples under both positive and negative ion modes, with highly consistent retention times and peak intensities (Figure 2A,B). This indicated that the detection signal was stable, the data were reliable, and the reproducibility was high throughout the analytical run. In the QC samples, more than 85% of the metabolites had CV values below 0.5, and more than 75% had CV values below 0.3, indicating good reproducibility of the experimental data (Figure 2C).

### 3.2. Overall Characteristics of Metabolites from Different Parts of C. huchouensis

**Figure 3:**
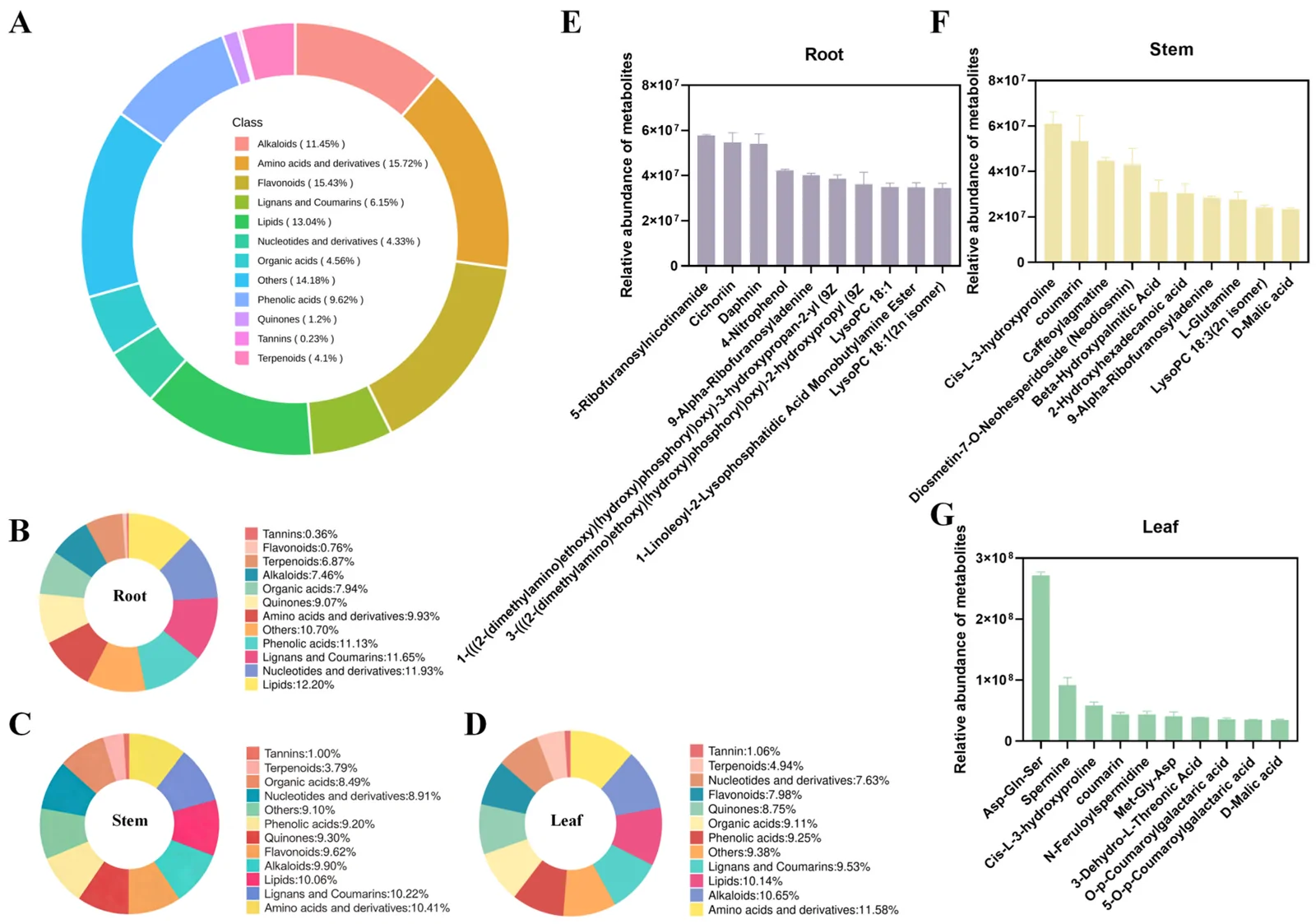
Metabolite distribution characteristics. (**A**) Identify the types and proportions of metabolites; (**B**) Proportion of metabolite content in roots of *C. huchouensis*; (**C**) Proportion of metabolite content in stems of *C. huchouensis*; (**D**) Proportion of metabolite content in leaves of *C. huchouensis.* (**E**) The top ten metabolites in terms of content in the roots of *C. huchouensis*; (**F**) The top ten metabolites in terms of content in the stems of *C. huchouensis*; (**G**) The top ten metabolites in terms of content in the leaves of *C. huchouensis*.

A total of 1561 metabolites were detected in the root, stem, and leaf samples of *C. huchouensis*. Among them, amino acids and their derivatives (15.72%), flavonoids (15.43%), lipids (13.04%), and alkaloids (11.45%) were relatively abundant, collectively accounting for 55.64% of the total metabolites (Figure 3A). *C. huchouensis* had high levels of lipids (12.20%), nucleotides and derivatives (11.93%), lignans and coumarins (11.65%), and phenolic metabolites (11.13%) in its roots, while the levels of flavonoids (0.76%) and tannins (0.36%) were low (Figure 3B). The content of amino acids and derivatives (10.41%), lignans and coumarins (10.22%), and lipids (10.06%) in the stem is relatively high, while the content of tannins (1.01%) is the lowest (Figure 3C). The content of amino acids and derivatives (11.58%), alkaloids (10.65%), and lipids (10.14%) in leaves is the highest, while the content of tannins (1.06%) is the lowest (Figure 3D). In the roots of *C. huchouensis*, the five most abundant metabolites were 5-ribofuranosylnicotinamide, cichoriin, daphnin, 4-nitrophenol, and 9-alpha-ribofuranosyladenine (Figure 3E). In the stems, the top five metabolites were cis-L-3-hydroxyproline, coumarin, caffeoylagmatine, neodiosmin, and β-hydroxypalmitic acid (Figure 3F). In the leaves, the five most abundant metabolites were Asp-Gln-Ser, spermine, cis-L-3-hydroxyproline, coumarin, and N-feruloylspermidine (Figure 3G).

### 3.3. Multivariate Statistical Analysis

**Figure 4:**
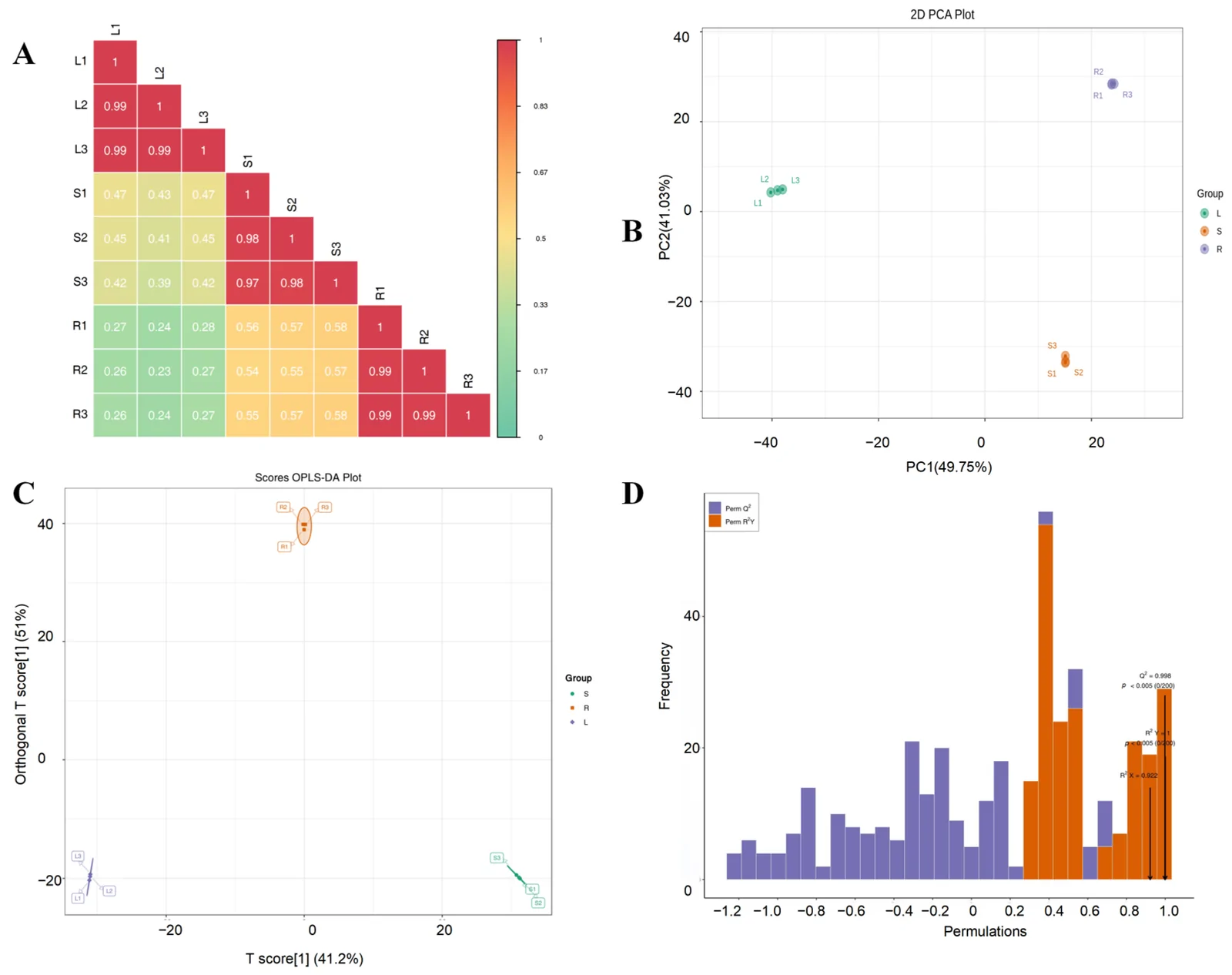
Multivariate statistical analysis. (**A**) Correlation diagram between samples; (**B**) PCA score plot; (**C**) OPLS-DA plot; (**D**) OPLS-DA validation diagram.

Metabolite differences among different organs of *C. huchouensis* were analyzed using ultra-high performance liquid chromatography–mass spectrometry (UPLC-MS). Correlation analysis indicated good biological reproducibility and consistency among samples within each group (Figure 4A). The principal component analysis (PCA) score plot revealed that the first principal component (PC1) and the second principal component (PC2) explained 49.75% and 41.02% of the total variance, respectively, cumulatively accounting for 90.77%. This high cumulative variance indicates substantial metabolite differences among the three groups (Figure 4B). The OPLS-DA also demonstrated clear separation of the three metabolite groups (Figure 4C). Furthermore, a high Q^2^ value (0.998) indicated that the model was well fitted and had strong predictive ability (Figure 4D).

### 3.4. Screening and Identification of Metabolites from Different Organs of C. huchouensis

**Figure 5:**
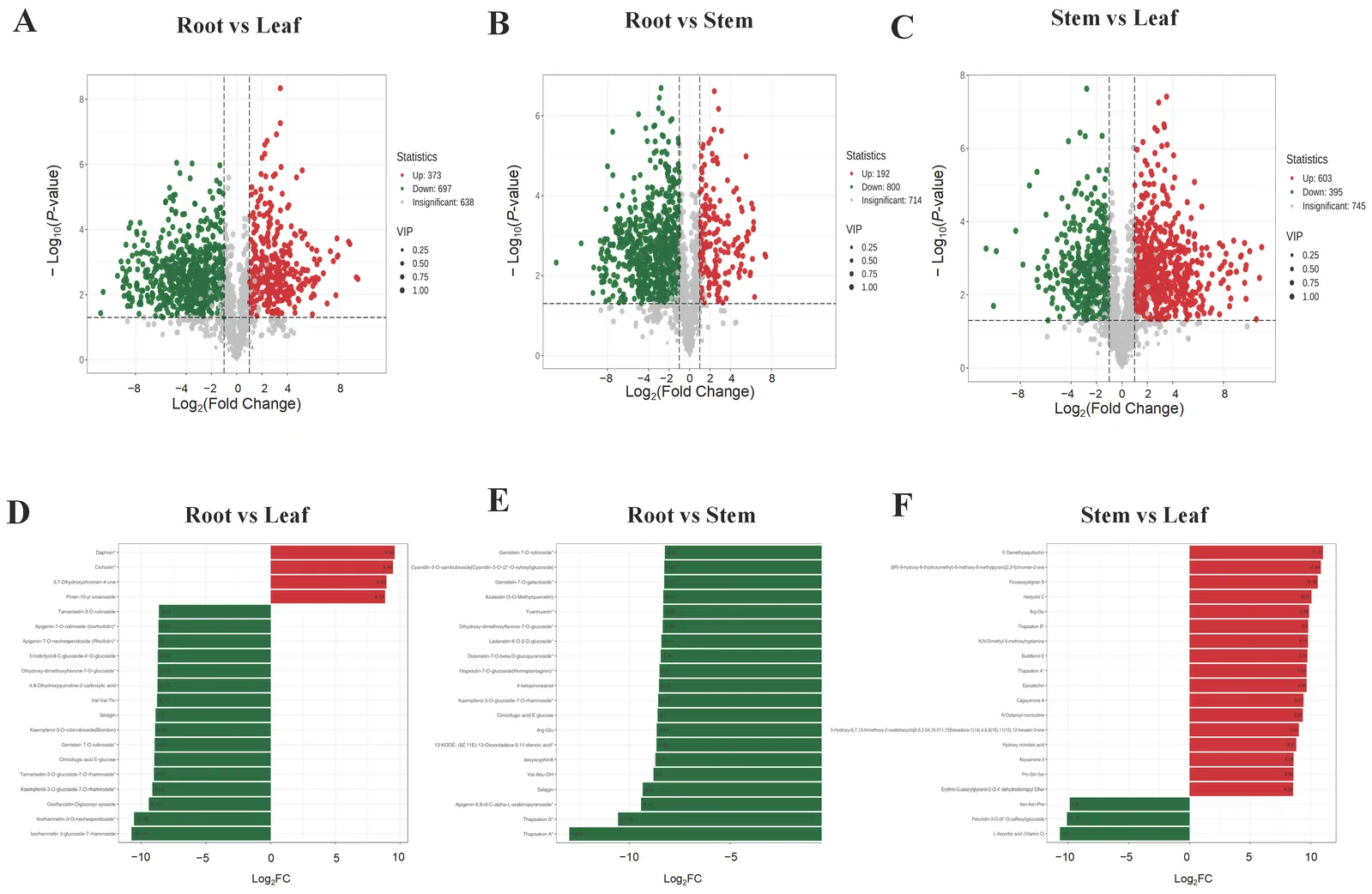
Metabolite analysis. (**A**) Volcano plot of DAMs in the root vs. leaf comparison. (**B**) Volcano plot of DAMs in the root vs. stem comparison. (**C**) Volcano plot of DAMs in the stem vs. leaf comparison. (**D**) Top 20 metabolites ranked by log_2_FC in the root vs. leaf comparison. (**E**) Top 20 metabolites ranked by log_2_FC in the root vs. stem comparison. (**F**) Top 20 metabolites ranked by log_2_FC in the stem vs. leaf comparison. Log_2_FC represents the log_2_-transformed fold change. Red indicates up-regulated metabolites, and green indicates down-regulated metabolites. The marked compounds (*) are isomers that cannot be distinguished by mass spectrometry. Different quantifier ions were selected for certain isomers during library construction, resulting in separate cumulative quantifications.

The criteria for screening differential metabolites (DAMs) using the OPLS-DA model were set as VIP ≥ 1 and FC ≥ 2 or ≤0.5. Compared with the root group, 1070 DAMs (373 up-regulated and 696 down-regulated) and 992 DAMs (192 up-regulated and 800 down-regulated) were identified in the leaf group and the stem group, respectively (Figure 5A,B). In the root vs. leaf comparison, among the top 20 metabolites with the greatest fold changes, two coumarins (daphnin and cichoriin), one chromone (3,7-dihydroxychromen-4-one), and one terpenoid (pinen-10-yl vicianoside) showed significantly higher accumulation in the roots, whereas 12 flavonoids, one quinone, one phenolic acid, one alkaloid, and one amino acid metabolite were significantly down-regulated (Figure 5C). In the root vs. stem comparison, among the top 20 DAMs, 12 flavonoids, two amino acids and their derivatives, two alkaloids, one phenolic acid, one lignan, one lipid, and one other metabolite were significantly down-regulated in the roots (Figure 5D). A total of 998 DAMs were detected in the stem vs. leaf comparison (603 up-regulated and 395 down-regulated) (Figure 5E). Compared with the leaf group, among the top 20 metabolites ranked by fold change, six lignans and coumarins, four alkaloids, two flavonoids, two terpenoids, two amino acids and their derivatives, and one lipid metabolite were significantly up-regulated in the stems, whereas one amino acid (Asn-Asn-Phe), one flavonoid (petunidin-3-O-(6″-O-caffeoyl)glucoside), and one other metabolite (L-ascorbic acid) were significantly down-regulated (Figure 5F).

### 3.5. KEGG Enrichment Analysis of DAMs

**Figure 6:**
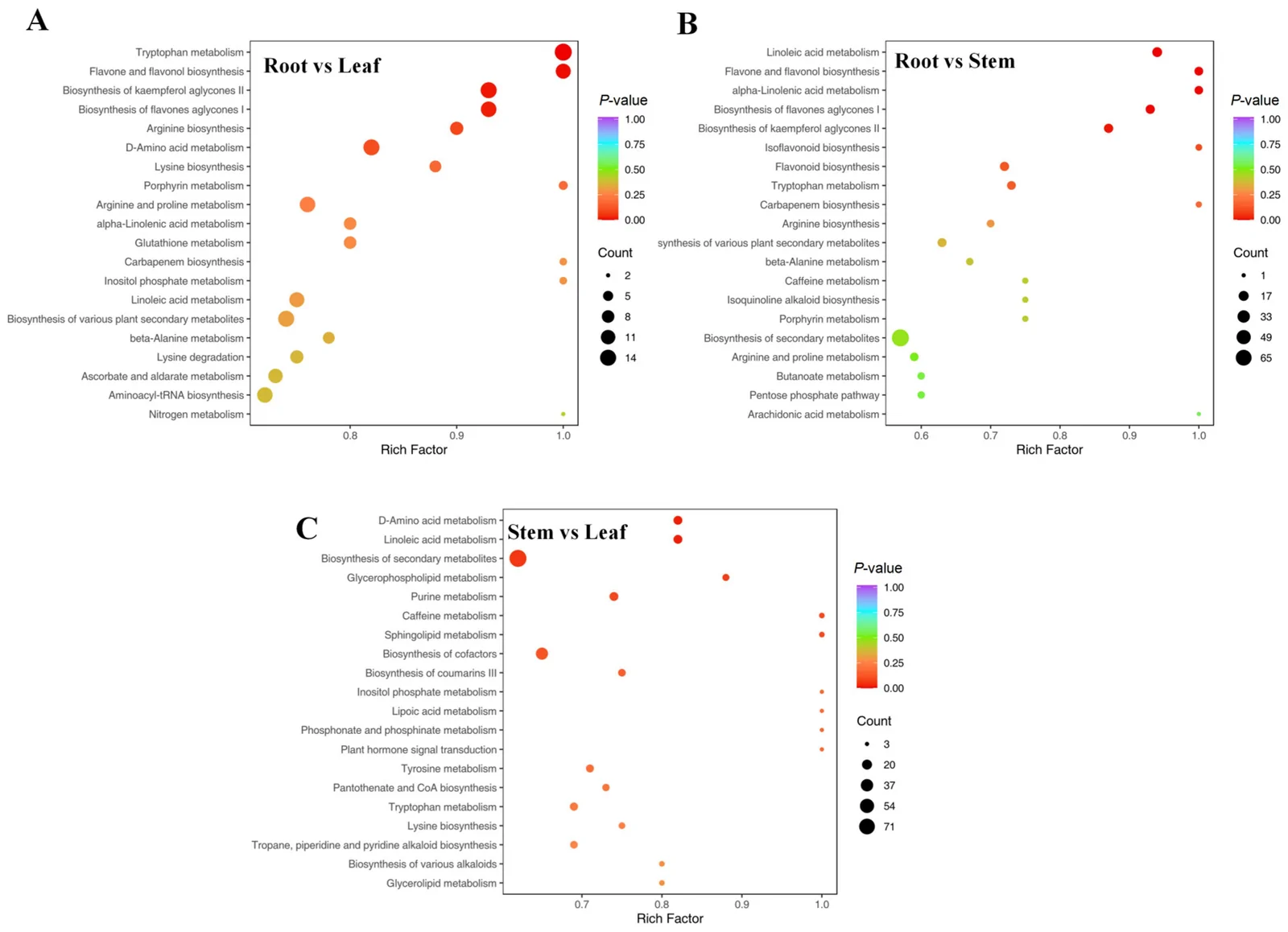
Metabolite analysis. KEGG enrichment plot of DAMs in different organs of *C. huchouensis*. (**A**) KEGG enrichment plot of DAMs in the root vs. leaf comparison. (**B**) KEGG enrichment plot of DAMs in the root vs. stem comparison. (**C**) KEGG enrichment plot of DAMs in the stem vs. leaf comparison. The color of the dots represents the *p*-value from the hypergeometric test; the color gradient ranges from purple to red, with more intense red indicating smaller *p*-values and thus higher statistical significance. The size of the dots denotes the number of differential metabolites in the corresponding pathway, with larger dots indicating a greater number of differential metabolites.

We performed pathway enrichment analysis on the significant DAMs in each comparison to identify the main pathways involved. In the root vs. leaf comparison, the significantly enriched pathways mainly included tryptophan metabolism (16 metabolites), flavone and flavonol biosynthesis (12 metabolites), biosynthesis of kaempferol aglycones II (14 metabolites), and biosynthesis of flavone aglycones I (13 metabolites) (Figure 6A). In the root vs. stem comparison, the significantly enriched pathways mainly comprised linoleic acid metabolism (16 metabolites), biosynthesis of flavone aglycones I (13 metabolites), biosynthesis of kaempferol aglycones II (13 metabolites), flavone and flavonol biosynthesis (11 metabolites), and α-linolenic acid metabolism (10 metabolites) (Figure 6B). In the stem vs. leaf comparison, the significantly enriched pathways included biosynthesis of secondary metabolites (87 metabolites), biosynthesis of cofactors (35 metabolites), D-amino acid metabolism (14 metabolites), and linoleic acid metabolism (14 metabolites) (Figure 6C).

### 3.6. Comparative Analysis of Relative Contents of Flavonoid Metabolites

**Figure 7:**
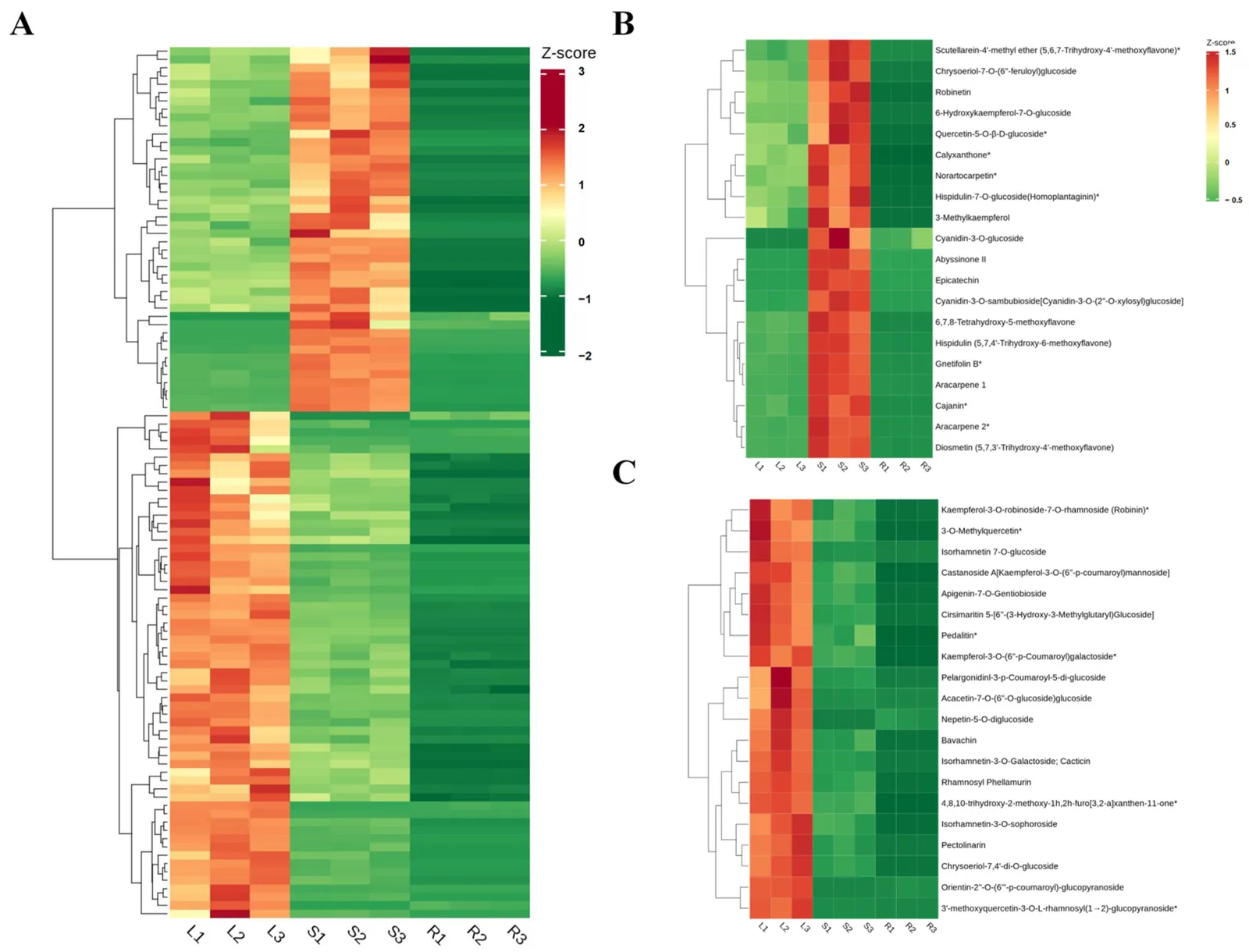
Differential flavonoid accumulation in leaves, stems, and roots of *C. huchouensis*. (**A**) Heatmap of all flavonoids showing significant differences among the three organs (leaves, stems, and roots). (**B**) Heatmap of the top 20 significantly up-regulated flavonoids in stems compared to leaves and roots. (**C**) Heatmap of the top 20 significantly up-regulated flavonoids in leaves compared to stems and roots. The Z-score color scale indicates relative metabolite abundance, with green representing lower abundance and red representing higher abundance. L1, L2, L3: leaves; S1, S2, S3: stems; R1, R2, R3: roots. The marked compounds (*) are isomers that cannot be distinguished by mass spectrometry. Different quantifier ions were selected for certain isomers during library construction, resulting in separate cumulative quantifications.

In this study, cluster heatmap analysis of the shared differential flavonoid metabolites among the roots, stems, and leaves revealed a significant tissue-specific accumulation pattern. A total of 105 significantly differentially accumulated flavonoid metabolites were identified across the three organs of *C. huchouensis*. The majority of flavonoids accumulated at significantly higher levels in the stems (44 flavonoids) and leaves (61 flavonoids), whereas their levels in the roots were generally low (Figure 7A). In the stems, the top 20 significantly up-regulated flavonoid metabolites included 7 flavones (diosmetin (5,7,3′-trihydroxy-4′-methoxyflavone), hispidulin (5,7,4′-trihydroxy-6-methoxyflavone), 6,7,8-tetrahydroxy-5-methoxyflavone, scutellarein-4′-methyl ether (5,6,7-trihydroxy-4′-methoxyflavone), chrysoeriol-7-*O*-(6″-feruloyl)glucoside, hispidulin-7-*O*-glucoside (homoplantaginin), and norartocarpetin); 5 flavonols (gnetifolin B, 6-hydroxykaempferol-7-*O*-glucoside, robinetin, 3-methylkaempferol, and quercetin-5-*O*-β-D-glucoside); 3 isoflavones (aracarpene 1, aracarpene 2, and cajanin); 2 anthocyanins (cyanidin-3-*O*-sambubioside [cyanidin-3-*O*-(2″-*O*-xylosyl)glucoside] and cyanidin-3-*O*-glucoside); 1 dihydroflavone (abyssinone II); 1 flavanol (epicatechin); and 1 other flavonoid (calyxanthone) (Figure 7B). In the leaves, the top 20 significantly up-regulated flavonoid metabolites included 8 flavones (orientin-2″-*O*-(6‴-p-coumaroyl)glucopyranoside, nepetin-5-*O*-diglucoside, pectolinarin, chrysoeriol-7,4′-di-*O*-glucoside, cirsimaritin 5-[6″-(3-hydroxy-3-methylglutaryl)glucoside], apigenin-7-*O*-gentiobioside, acacetin-7-O-(6″-*O*-glucoside)glucoside, and pedalitin); 8 flavonols (3′-methoxyquercetin-3-*O*-L-rhamnosyl(1→2)glucopyranoside, isorhamnetin-3-*O*-galactoside (cacticin), isorhamnetin 7-*O*-glucoside, kaempferol-3-*O*-(6″-p-coumaroyl)galactoside, isorhamnetin-3-*O*-sophoroside, castanoside A [kaempferol-3-*O*-(6″-p-coumaroyl)mannoside], kaempferol-3-*O*-robinoside-7-*O*-rhamnoside (robinin), and 3-*O*-methylquercetin); 1 dihydroflavonol (rhamnosyl phellamurin); 1 anthocyanin (pelargonidin-3-p-coumaroyl-5-di-glucoside); 1 dihydroflavone (bavachin); and 1 other flavonoid (4,8,10-trihydroxy-2-methoxy-1H,2H-furo[3,2-a]xanthen-11-one) (Figure 7C).

### 3.7. Comparative Analysis of Relative Contents of Amino Acids

**Figure 8:**
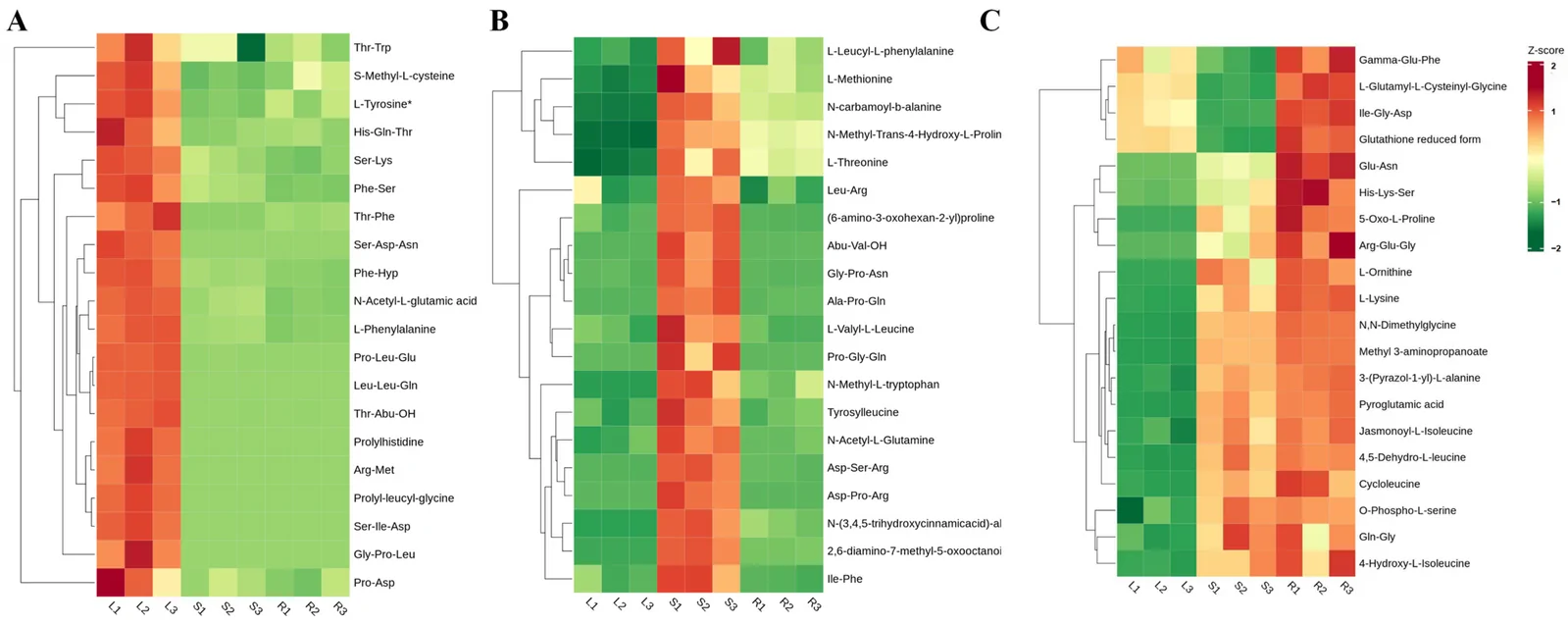
Differential amino acid accumulation in leaves, stems, and roots of *C. huchouensis*. (**A**) Heatmap of the top 20 significantly up-regulated amino acids in leaves compared to stems and roots. (**B**) Heatmap of the top 20 significantly up-regulated amino acids in stems compared to leaves and roots. (**C**) Heatmap of the top 20 significantly up-regulated amino acids in roots compared to leaves and stems. The Z-score color scale indicates relative metabolite abundance, with green representing lower abundance and red representing higher abundance. L1, L2, L3: leaves; S1, S2, S3: stems; R1, R2, R3: roots. The marked compounds (*) are isomers that cannot be distinguished by mass spectrometry. Different quantifier ions were selected for certain isomers during library construction, resulting in separate cumulative quantifications.

To explore the accumulation characteristics of amino acid metabolites in different organs of *C. huchouensis*, the differential amino acids in leaves, stems, and roots were compared. A total of 127 significantly differentially expressed amino acid-related metabolites were identified across three organs of *C. huchouensis*. Significantly higher accumulation of the majority of metabolites occurred in leaves (46 amino acids) and stems (68 amino acids), in contrast to their generally low levels in roots (Figure S1). The results revealed 20 amino acids and their derivatives with significantly high abundance across the three organs. Peptides and modified amino acids, including Thr-Trp, S-methyl-L-cysteine, L-tyrosine, His-Gln-Thr, Ser-Lys, Phe-Ser, Thr-Phe, Ser-Asp-Asn, Phe-Hyp, and N-acetyl-L-glutamic acid, accumulated at markedly higher levels in the leaves (Figure 8A). These metabolites were predominantly dipeptides, tripeptides, and acetylated or methylated derivatives, suggesting active protein turnover and secondary metabolic processes in the leaves. Amino acids such as L-leucyl-L-phenylalanine, L-methionine, N-carbamoyl-β-alanine, N-methyl-trans-4-hydroxy-L-proline, L-threonine, and Leu-Arg were significantly enriched in the stems (Figure 8B). In the roots, amino acids such as γ-Glu-Phe, L-glutamyl-L-cysteinylglycine, Ile-Gly-Asp, reduced glutathione (GSH), Glu-Asn, His-Lys-Ser, 5-oxo-L-proline, and Arg-Glu-Gly were significantly enriched (Figure 8C).

### 3.8. Comparative Analysis of Relative Contents of Phenolic Acids

**Figure 9:**
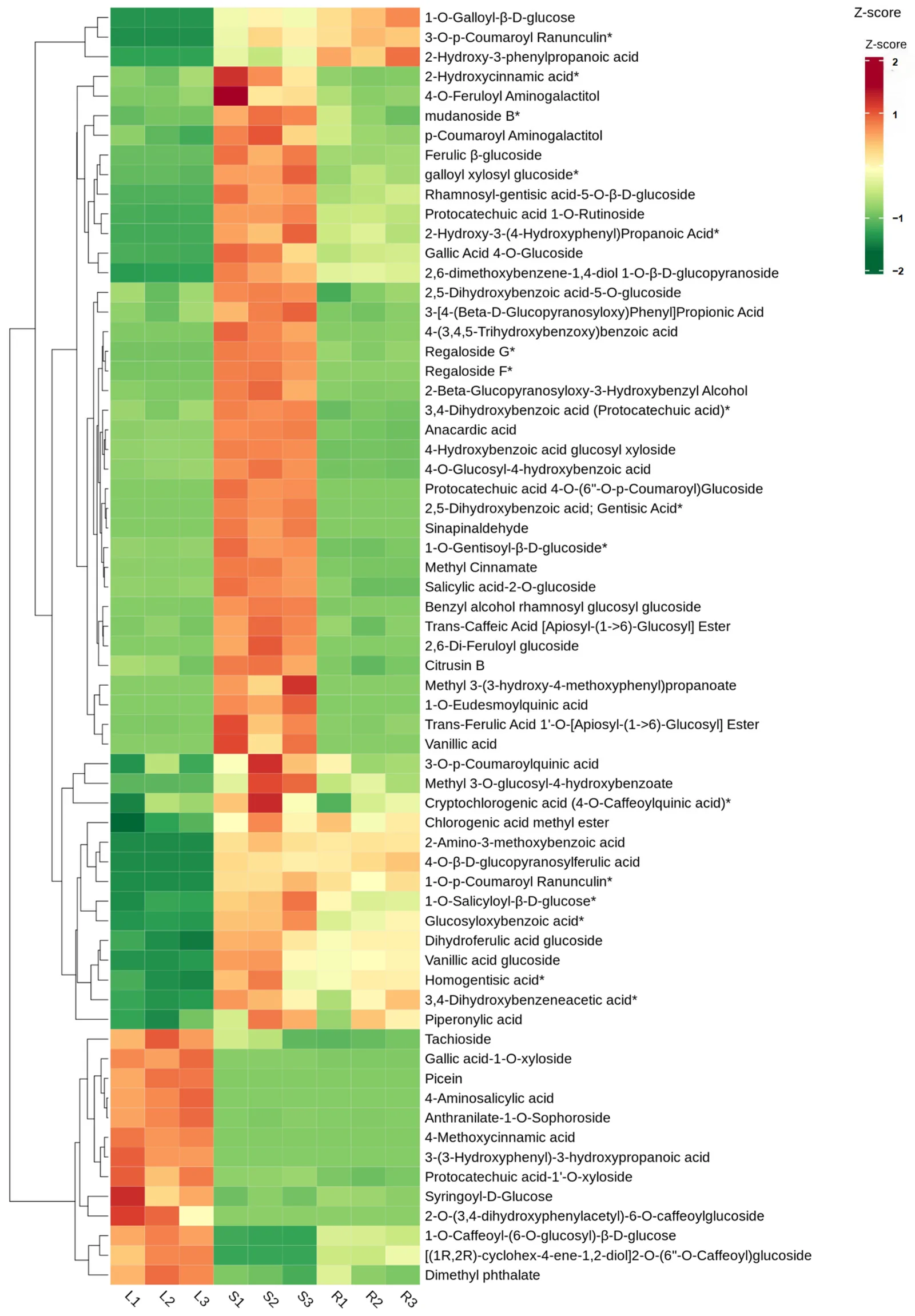
Differential accumulation of phenolic acids in leaves, stems, and roots of *C. huchouensis*. The Z-score color scale indicates relative metabolite abundance, with green representing lower abundance and red representing higher abundance. L1, L2, L3: leaves; S1, S2, S3: stems; R1, R2, R3: roots. The marked compounds (*) are isomers that cannot be distinguished by mass spectrometry. Different quantifier ions were selected for certain isomers during library construction, resulting in separate cumulative quantifications.

A total of 65 significantly differentially expressed phenolic acid metabolites were detected in the three organs of *C. huchouensis* (Figure 9). Thirteen phenolic acid metabolites accumulated at significantly higher levels in the leaves, whereas their content remained relatively low in the stems and roots. These leaf-enriched metabolites included tachioside, gallic acid-1-O-xyloside, picein, 4-aminosalicylic acid, anthranilate-1-O-sophoroside, 4-methoxycinnamic acid, 3-(3-hydroxyphenyl)-3-hydroxypropanoic acid, protocatechuic acid-1′-O-xyloside, syringoyl-D-glucose, and 2-O-(3,4-dihydroxyphenylacetyl)-6-O-caffeoylglucoside, among others.

Forty-nine phenolic acid metabolites accumulated predominantly in the stems of *C. huchouensis*, mainly including 2-hydroxycinnamic acid, 4-*O*-feruloyl aminogalactitol, mudanoside B, feruloyl β-glucoside, galloyl xylosyl glucoside, gallic acid 4-*O*-glucoside, regaloside G, regaloside F, sinapinaldehyde, and methyl cinnamate.

In the roots of *C. huchouensis*, the significantly enriched phenolic acid metabolites were 1-*O*-galloyl-β-D-glucose, 3-*O*-*p*-coumaroylranunculin, and 2-hydroxy-3-phenylpropanoic acid.

### 3.9. Comparative Analysis of Relative Contents of Other Secondary Metabolites

The cluster heatmap clearly revealed the differential accumulation patterns of 56 common alkaloid metabolites shared among the roots, stems, and leaves across different organs. Overall, the accumulation trends in leaves and stems were highly similar, whereas a clear separation was observed between roots and aboveground parts. This organ-specific distribution pattern reflects the spatial allocation and functional differentiation of alkaloids within the plant (Figure S2). A large number of alkaloids were significantly up-regulated in the leaves (32 alkaloids), followed by the stems (21 alkaloids), indicating that the aboveground organs are the primary sites for alkaloid biosynthesis and accumulation. In contrast, roots showed a distinctly opposing trend to leaves and stems, with the majority of alkaloids displaying significantly reduced abundance in roots. Only three alkaloids (ailanindole, pyrrolidine-2-carboxamide, and caffeine) showed specifically high expression in roots (Figure S2).

**Figure 10:**
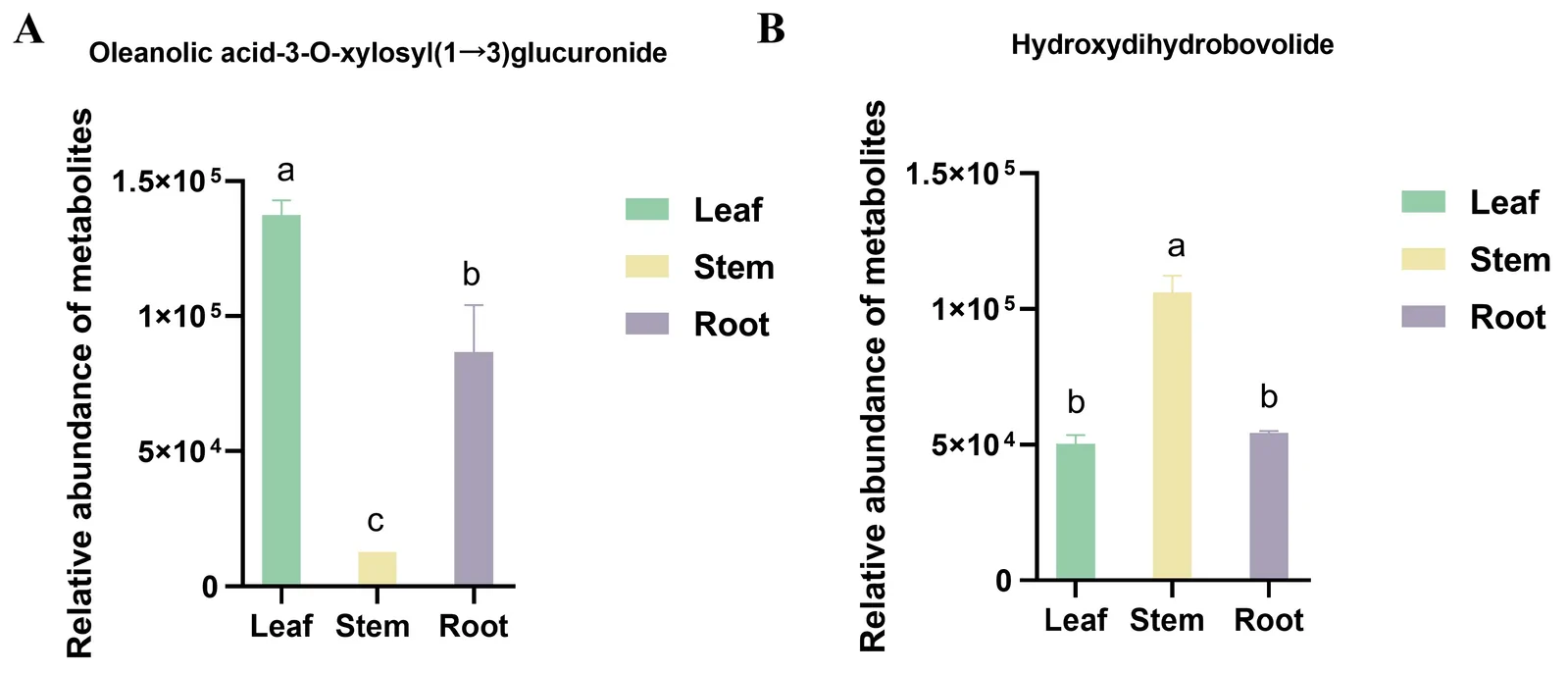
Relative contents of oleanolic acid-3-O-xylosyl(1→3)glucuronide and hydroxydihydrobovolide in the leaves, stems, and roots of *C. huchouensis*. (**A**) Oleanolic acid-3-O-xylosyl(1→3)glucuronide. (**B**) Hydroxydihydrobovolide. Lowercase letters denote significant differences (*p* < 0.05) based on one-way ANOVA with Duncan’s multiple comparison test.

One differential triterpenoid saponin, oleanolic acid-3-*O*-xylosyl(1→3)glucuronide, and one lactone metabolite, hydroxydihydrobovolide, were detected in the roots, stems, and leaves of *C. huchouensis* (Figure 10). The relative content of oleanolic acid-3-*O*-xylosyl(1→3)glucuronide was highest in the leaves, being 10.86-fold higher than in the stems and 1.58-fold higher than in the roots (Figure 10A). The relative content of hydroxydihydrobovolide was highest in the stems, being 2.12-fold higher than in the leaves and 1.95-fold higher than in the roots; it was significantly higher in the stems than in both leaves and roots, whereas no significant difference was observed between leaves and roots (Figure 10B).

## 4. Discussion

A large number of significantly differentially expressed flavonoid compounds were detected in the stems, and many of them have been confirmed to possess remarkable pharmacological activities. Robinetin not only inhibits HIV-1 integrase and acetylcholinesterase, demonstrating potent antiviral activity, but also exhibits moderate antiproliferative activity in cancer cell lines [17]. 3-Methylkaempferol, isolated from the medicinal plant *Psiadia dentata*, has been confirmed as an effective inhibitor of poliovirus type 2 replication [18]. Hispidulin triggers reactive oxygen species (ROS)-mediated apoptosis in human non-small cell lung cancer cells via the endoplasmic reticulum stress pathway [19]. Diosmetin alleviates hyperuricemic nephropathy by activating the BNIP3/Nrf2/GPX4 pathway to inhibit ferroptosis of renal tubular epithelial cells induced by monosodium urate crystals [20]. Cyanidin-3-*O*-glucoside exhibits anti-PRRSV activity [21], while cajanin, a natural small-molecule compound, suppresses osteoclastogenesis and bone resorption both in vitro and in animal models, thereby alleviating pathological bone loss [22]. In addition, bavachin, which was specifically enriched in the leaves, can alleviate rheumatoid arthritis by suppressing the NF-κB signaling pathway and angiogenesis [23], while pedalitin, in combination with amphotericin B, exhibits potent synergistic anticryptococcal activity against *Cryptococcus neoformans* [24].

Among the amino acid pool identified in the stems, L-tyrosine and L-phenylalanine function as precursors for phenylpropanoid metabolism and flavonoid biosynthesis [25]. The sulfur-containing amino acid S-methyl-L-cysteine has been shown to possess significant pharmacological activities, including antioxidant, anti-inflammatory, and insulin-resistance-improving effects [26]. N-carbamoyl-L-glutamic acid has been approved by the U.S. Food and Drug Administration (FDA) for the treatment of fatal hyperammonemia [27].

Among the amino acids specifically enriched in the leaves, L-methionine, a sulfur-containing amino acid, serves as a core component of the plant heavy metal detoxification network and also as the precursor of S-adenosyl-L-methionine, the principal methyl donor in living organisms, thus contributing to the prevention of heavy metal-induced hepatotoxicity [28]. N-Methyl-trans-4-hydroxy-L-proline also accumulated at high levels in the stems. Studies have demonstrated that it can significantly reduce TNF-α levels and inhibit the activation of nuclear factor-κB (NF-κB), exhibiting potent anti-inflammatory effects [29].

Of particular note is the extremely high accumulation of glutathione and its precursors, γ-Glu-Phe and L-glutamyl-L-cysteinylglycine, in the roots, a phenomenon also observed in rice and alfalfa [30,31]. The remarkably high glutathione levels in roots reflect their role as the primary stress-responsive organ, participating in responses to heavy metal and oxidative stress [30]. 5-Oxo-L-proline also exhibits significant antioxidant activity [32]. The accumulation of these amino acids reflects a robust antioxidant system in the roots of *C. huchouensis*, suggesting enhanced antioxidant capacity and detoxification potential in this organ.

The phenolic acid components enriched in the leaves also exhibit pharmacological values that should not be overlooked. Among them, tachioside has been attributed with both anti-inflammatory and potent antioxidant activities [33], whereas antioxidant and neuroprotective potential are associated with picein [34]. As a classic anti-tuberculosis drug, 4-aminosalicylic acid exerts anti-mycobacterial effects against *Mycobacterium tuberculosis* [35]. Turning to the stems, their phenolic acid composition exhibits a distinct functional bias. 2-Hydroxycinnamic acid displays significant antibacterial activity against *Staphylococcus aureus* [36] and also exhibits distinct neuroprotective properties relevant to Alzheimer’s disease [37]. Methyl cinnamate can activate the CAMKK2-AMPK signaling pathway, thereby inhibiting the differentiation of 3T3-L1 adipocytes and lipid accumulation in HepG2 cells, ultimately reducing triglyceride levels [38].

Although the phenolic acids in the roots are relatively limited in variety, the pharmacological targeting of their individual constituents is equally important. Among these, 1-*O*-galloyl-β-D-glucose functions as an aldose reductase inhibitor and is considered a promising candidate for the treatment of diabetes [39].

Notably, colchicoside, trigonelline, dopamine, and vasicinone, which accumulated at high levels in the leaves, possess well-defined pharmacological activities. Colchicoside is an anti-gout agent that inhibits microtubule polymerization. This action subsequently blocks neutrophil chemotaxis and the release of inflammatory factors [40]. Trigonelline exhibits hypoglycemic, lipid-lowering, neuroprotective, and antidepressant effects [41,42,43]. Dopamine is a powerful vasoactive medication indicated for hypotension induced by cardiogenic shock or heart failure [44,45]. Vasicinone alleviates asthma and bronchitis by relaxing the airways [46,47]. Similarly, 3-indolepropionic acid, stachydrine, tryptamine, and *N*-*cis*-cinnamoylagmatine, which accumulated at high levels in the stems, each possess important functional activities. By scavenging hydroxyl radicals, 3-indolepropionic acid produces neuroprotective effects and has demonstrated efficacy in models of Alzheimer’s and Parkinson’s diseases [48]. Stachydrine, the main active component of *Leonurus japonicus*, exhibits diuretic, anti-fibrotic, and renal protective effects [49]. Tryptamine is involved in neuromodulation, antioxidation, and immunomodulation [50,51]. *N*-*cis*-Cinnamoylagmatine lowers postprandial blood glucose through α-glucosidase inhibition, the same target of the hypoglycemic agent acarbose [52]. Only three alkaloids exhibited specifically high expression in the roots. Among them, caffeine exerts its effects by inhibiting phosphodiesterase 4 to prevent the degradation of cyclic adenosine monophosphate, thereby serving as an adjunct therapy for asthma and chronic obstructive pulmonary disease, while also exhibiting multiple activities including diuretic, antitumor, antioxidant, and skin-protective effects [53].

Oleanolic acid-3-*O*-xylosyl(1→3)glucuronide is a glycosylated derivative of oleanolic acid and belongs to the oleanane-type triterpenoid saponins. Previous studies have shown that oleanolic acid and its glycoside derivatives exhibit broad-spectrum antitumor effects as well as immunomodulatory activities [54,55]. Studies have identified hydroxydihydrobovolide as a key lactone in Portulaca oleracea that displays cytotoxicity and markedly suppresses the proliferation of the human neuroblastoma SH-SY5Y cell line [56]. Furthermore, hydroxydihydrobovolide isolated from *Litsea verticillata* has been shown to inhibit HIV, demonstrating significant antiviral potential [56].

*C. huchouensis* and *C. hexapetala* share similar chemical profiles, as both are rich in flavonoids and phenolic compounds [57]. Research indicates that these active components exert a marked hypouricemic effect in mouse models of hyperuricemia, while also mitigating renal histopathological injury [57]. The roots of *C. chinensis* are rich in triterpenoid saponins with novel structural features, which have been demonstrated to exert protective effects on articular cartilage and to effectively suppress local inflammatory responses [9,10]. It is noteworthy that in the mainstream medicinal species of *Clematis*, such as *C. chinensis* and *Clematis terniflora*, the pharmacologically active constituents are predominantly concentrated in the triterpenoid saponins of the roots [9,58]. In contrast, the signature saponin of *C. huchouensis*, namely oleanolic acid-3-O-xylosyl(1→3)glucuronide, exhibits the highest accumulation in the leaves, followed by the roots. A large number of alkaloids have been isolated from the rhizomes of *C. chinensis* [59]. However, a substantial number of highly expressed alkaloids, such as colchicine, trigonelline, and dopamine, were also detected in the stems and leaves of *C. huchouensis*. In marked contrast, the roots exhibited considerably lower alkaloid levels, where only three specifically highly expressed alkaloids (ailanindole, pyrrolidine-2-carboxamide, and caffeine) were detectable. This indicates that the alkaloid resources of this species are predominantly distributed in the above-ground parts. Taken together, these differential distribution patterns suggest that whereas the traditional medicinal use of *Clematis* is limited to the roots, the stems and leaves of *C. huchouensis* harbor a diverse array of characteristic bioactive constituents, which provides a chemical rationale for the folk practice of utilizing the whole plant for medicinal purposes.

The results of the present study reveal significant metabolite variations among different organs of *C*. *huchouensis*. Nevertheless, such variations are not governed solely by genetic factors. Other factors, including environmental conditions, cultivation practices and developmental stages, may also exert substantial influences on the accumulation of secondary metabolites.

Numerous studies have investigated the effects of environmental factors on the secondary metabolic processes of *Clematis* species. Ashrafzadeh found that the contents of total phenolics, total flavonoids, and total alkaloids in *C. isfahanica* under semi-arid climatic conditions were significantly higher than those in plants from arid regions, and that soil pH, electrical conductivity, and total nitrogen content were all positively correlated with metabolite accumulation [60]. Zhang further confirmed that the suitable distribution areas of *C. tangutica* on the Qinghai–Tibet Plateau are primarily determined by seasonal UV-B radiation and altitude, with flavonoids and phenolic acids reaching peak accumulation at elevations of 2500–3500 m, revealing a coupling mechanism between environmental stress and secondary metabolic pathways [61]. Collectively, these lines of evidence suggest that the distinctive metabolic profiles of *C. huchouensis*, a geo-authentic herb from Huzhou, may be closely associated with the site-specific hydrological, edaphic, and light conditions of its producing area.

Developmental stage is also a key factor affecting secondary metabolite accumulation. In *C. isfahanica*, total phenolics, flavonoids, and alkaloids were significantly higher at flowering than during vegetative and mature stages [60]. To minimize developmental variation, all samples in this study were collected during vigorous vegetative growth in June. However, future time-series sampling is still needed to fully assess the dynamic influence of phenological phases on metabolic profiles.

Cultural practices may likewise affect the accumulation of secondary metabolites. Moderate pruning (50%) combined with flower removal is an effective cultivation regulation strategy that can directionally promote the accumulation of bioactive isoflavonoids without compromising root biomass or astragaloside IV content, thereby optimizing the medicinal quality of Astragalus membranaceus var. Mongholicus [62]. The content of taiwanianone A in the tubers of *Cynanchum taiwanianum* increases with rising potassium fertilizer application, but is reduced by excessive nitrogen fertilization [63].

The wild samples used in this study represent the metabolic traits of the Huzhou Authentic Production Area Specific Habitat. Future work should combine multi-region comparisons, time-series sampling, and controlled cultivation to better understand how genotype–environment interactions shape the metabolic profiles and medicinal quality of *C. huchouensis*.

## 5. Conclusions

This study firstly performed widely targeted metabolomic analysis on *C. huchouensis* from the Huzhou authentic producing area. A total of 1561 metabolites were identified in roots, stems and leaves, showing obvious organ-specific accumulation characteristics. Flavonoids, alkaloids and most phenolic acids were mainly enriched in leaves and stems, while roots accumulated large amounts of glutathione and its precursors, presenting a strong antioxidant and stress defense system. Metabolic differences among organs were mainly involved in flavonoid biosynthesis, linoleic acid metabolism and other key pathways. A variety of metabolites with anti-inflammatory, antitumor, antiviral and other pharmacological activities showed significant tissue-specific distribution. These results revealed the metabolic basis of its medicinal value and provided scientific support for the resource evaluation, quality control and rational utilization of *C. huchouensis*.

## Data Availability

The original contributions presented in this study are included in the article. Further inquiries can be directed to the corresponding author.

## Supplementary Materials

The following supporting information can be downloaded at: https://www.mdpi.com/article/10.3390/metabo16080561/s1, Figure S1. Differential accumulation of amino acids in leaves, stems, and roots of *C. huchouensis*. The Z-score color scale indicates relative metabolite abundance, with green representing lower abundance and red representing higher abundance. L1, L2, L3: leaves; S1, S2, S3: stems; R1, R2, R3: roots. Figure S2. Differential accumulation of alkaloid in leaves, stems, and roots of *C. huchouensis*. The Z-score color scale indicates relative metabolite abundance, with green representing lower abundance and red representing higher abundance. L1, L2, L3: leaves; S1, S2, S3: stems; R1, R2, R3: roots.
